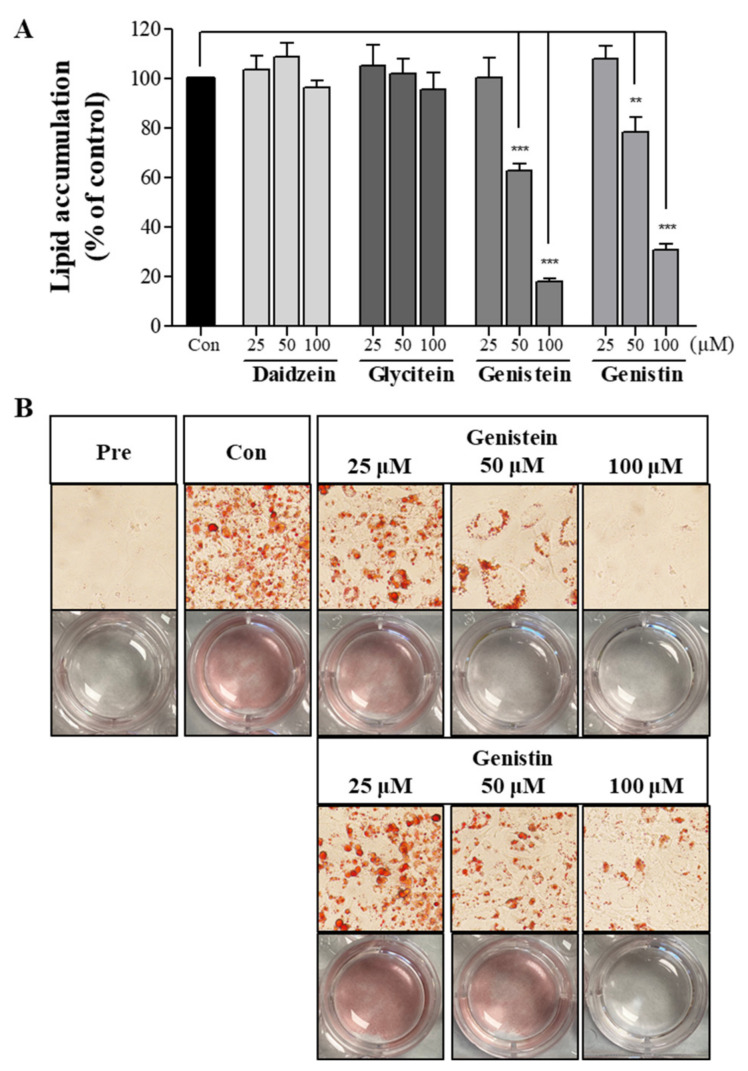# Correction: Choi et al. Genistin: A Novel Potent Anti-Adipogenic and Anti-Lipogenic Agent. *Molecules* 2020, *25*, 2042

**DOI:** 10.3390/molecules29030714

**Published:** 2024-02-04

**Authors:** Yae Rim Choi, Jaewon Shim, Min Jung Kim

**Affiliations:** 1 Research Division of Food Functionality, Korea Food Research Institute, Wanju 55365, Republic of Korea; uiu7895@naver.com (Y.R.C.); jwshim@kfri.re.kr (J.S.); 2 Department of Food Science and Engineering, Ewha Womans University, Seoul 03760, Republic of Korea

**Error in Figure**

In the original publication [1], there was a mistake in **Figure 2B**. **Among the uploaded Oil Red O staining images in Figure 2B, genistein and genistin 100 μM were used identically to the images of genistein and genistin 50 μM, but this was an error in the process of inserting images from the same 12-well plate, so this part was corrected.** The corrected **Figure 2** appears below. The authors apologize for any inconvenience caused and state that the scientific conclusions are unaffected. This correction was approved by the Academic Editor. The original publication has also been updated.